# A Synthetic Bandwidth Method for High-Resolution SAR Based on PGA in the Range Dimension

**DOI:** 10.3390/s150715339

**Published:** 2015-06-29

**Authors:** Jincheng Li, Jie Chen, Wei Liu, Pengbo Wang, Chunsheng Li

**Affiliations:** 1 School of Electronic and Information Engineering, Beihang University, Beijing 100191, China; E-Mails: lijincheng_buaa@163.com (J.L.); chenjie@buaa.edu.cn (J.C.); lics@buaa.edu.cn (C.L.); 2 Electronic and Electronic Engineering Department, University of Sheffield, Sheffield S1-3JD, UK; E-Mail: w.liu@sheffield.ac.uk

**Keywords:** synthetic bandwidth, synthetic aperture radar (SAR), ultra-high range resolution

## Abstract

The synthetic bandwidth technique is an effective method to achieve ultra-high range resolution in an SAR system. There are mainly two challenges in its implementation. The first one is the estimation and compensation of system errors, such as the timing deviation and the amplitude-phase error. Due to precision limitation of the radar instrument, construction of the sub-band signals becomes much more complicated with these errors. The second challenge lies in the combination method, that is how to fit the sub-band signals together into a much wider bandwidth. In this paper, a novel synthetic bandwidth approach is presented. It considers two main errors of the multi-sub-band SAR system and compensates them by a two-order PGA (phase gradient auto-focus)-based method, named TRPGA. Furthermore, an improved cut-paste method is proposed to combine the signals in the frequency domain. It exploits the redundancy of errors and requires only a limited amount of data in the azimuth direction for error estimation. Moreover, the up-sampling operation can be avoided in the combination process. Imaging results based on both simulated and real data are presented to validate the proposed approach.

## 1. Introduction

Synthetic aperture radar (SAR) is a powerful active remote sensing system with all-time and all-weather observation ability to form two-dimensional high-resolution images [1–3]. The high resolution in the range dimension can be obtained by enhancement of signal bandwidth. To form a larger bandwidth, a synthetic bandwidth technique has been applied in several existing SAR systems, such as PAMIR, RAMES, ROOFSAR and RADARSAT2 [4–7]. In these multi-sub-band systems, the synthetic bandwidth technique can lower the demand on radar hardware at the expense of increased system complexity, posing many challenges for SAR signal processing.

For a practical SAR system, the linearity of the transmitted linear frequency-modulated (LFM) signal cannot be achieved exactly due to instrument errors and hardware limitations in implementing the flat amplitude-phase characteristics of the transmitting and receiving loops. Furthermore, sub-band partition requires synchronization between the radar subsystems, and even a small timing error can impair phase coherence and result in loss in range resolution. To solve the problem, the relative calibration approach was proposed in [8], where the timing deviation is extracted from the internal calibration data. However, only the timing error between the subsystems is considered there, and the filter errors are neglected. Hu *et al.* proposed an improved two-step method in [9], which employs the relative calibration approach as the preliminary step, and the residual phase errors are modelled as frequency-domain polynomials to be estimated based on the image contrast. A disadvantage of this method is large computational load, since iteration is required to calculate the polynomials of the phase errors within and between the sub-bands; moreover, it neglects the compensation of the amplitude error of the spectrum, which will reduce image quality. The method presented in [10] considers both amplitude and phase errors and constructs a correction filter by measuring the spectrum of a real physical target in the scene. However, the accuracy of the method is dependent on the choice of the target analysed, and it lacks robustness, since only one strong point target is utilized in the application. Other than the estimation and compensation of the foregoing system errors, another challenge lies in the combination method. The sub-band signals can be combined either in the time domain or the frequency domain. A time-domain method was proposed by [11] in which the sub-band signal is required to be up-sampled first to avoid aliasing. This will increase the computational load significantly, given the required pre-processing steps, including frequency shift, phase correction and time shift. To avoid the up-sampling operation, a frequency-domain combination method was presented in [6], which is represented as a cut-paste method. However, the specific design of the radar parameters is needed for this method, which will increase the system complexity.

In this paper, a novel bandwidth construction method is proposed to tackle the aforementioned problems, where the system errors are divided into two orders and compensated by a two-order phase gradient auto-focus (PGA)-based method, which we call TRPGA. The first-order errors, including the timing error and most of the amplitude-phase errors, are extracted from the internal calibration data. In second-order error correction, we implement the weighted PGA (WPGA) in the range direction to compensate the residual filter error outside the internal loop. Moreover, an improved cut-paste combination approach is presented without the need of up-sampling and parameter adjustment. The proposed method is only applied in the range dimension and has no effect on the signal characteristics in the azimuth dimension, making it suitable for any SAR operating mode, such as strip-map, spotlight, ScanSAR, *etc*. The validity of the proposed method is verified by imaging results based on both simulated and real SAR data.

This paper is organized as follows: In Section 2, the signal and error model of the multi-sub-band system are presented. In Section 3, the effect of the timing error is analysed, and the upper limit of the timing error is also derived. The related works of the proposed method, including internal calibration and the PGA technique, are reviewed in Section 4, and the proposed construction method is developed in Section 5. Imaging results on both simulated and real SAR raw data are provided in Section 6, and conclusions are drawn in Section 7.

## 2. Signal and Error Model of the Multi-Sub-Band System

### 2.1. Signal Model

**Figure 1:**
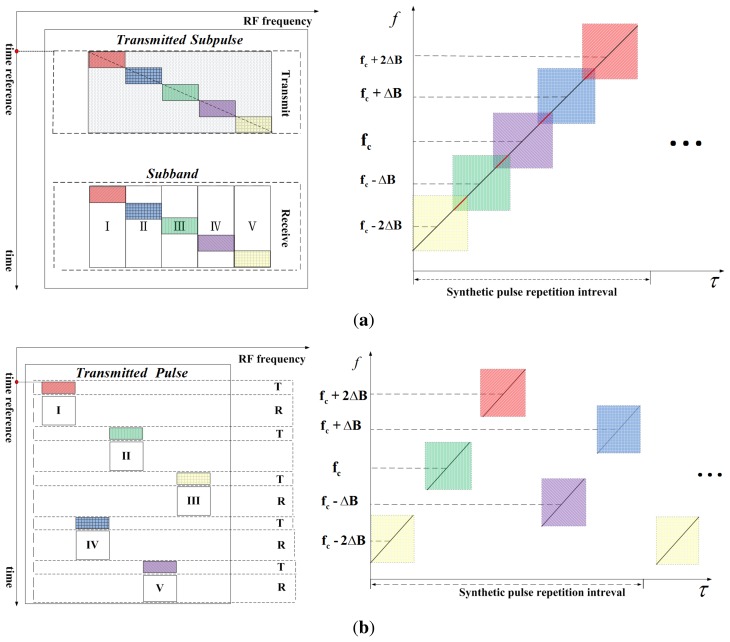
Schematic diagram and time-frequency diagram (TFD) in the range direction of two operation modes of a multi-sub-band system. (**a**) Consecutive stepped-frequency mode; (**b**) non-consecutive stepped-frequency mode.

Currently, the synthetic bandwidth technique can be implemented in two modes. The schematic diagram and time-frequency diagram (TFD) in the range direction of the two modes are shown in Figure 1, where the number of sub-bands is five. In Figure 1a, the system transmits one wide-bandwidth signal or several successive chirp signals, and the transmitted signals are received as successive chirp signals offset by a certain step frequency. This is referred to as the consecutive stepped-frequency approach. The other method is described as non-consecutive stepped-frequency mode and is sketched in Figure 1b, where the transmitted and received signals are composed of several non-consecutive chirps in different sub-bands to avoid range ambiguity.

As an asynchronous system, the time reference should be unified for different sub-bands. Without loss of generality, the range time reference of all of the sub-bands is defined as the initial time of the transmitted signal in the first sub-band, as shown in Figure 1. Assuming the sub-band number is *N* and *k* is the transmitting order of the sub-band signal, the transmitted signal of the sub-band can be expressed as:

(1) $$s_{t}(\tau ,k)=\mathrm{rect}\left(\frac{\tau -\Delta t(k)}{T/N}\right)\;\exp\;\{j2\pi f_{c}(k)(\tau -\Delta t(k))+j\pi K_{r}{\left(\tau -\Delta t(k)\right)}^{2}\},k=1,2,\cdots ,N$$

where *τ* is the fast time in the range dimension, *T* is the total pulse length of the synthesized signal, *f_c_* (*k*) represents the centre frequency of the *k*-th sub-band, *K_r_* denotes the linear FM rate and Δ*t* (*k*) is the deterministic time delay of the *k*-th sub-band compared with the first sub-band. For simplicity, the transmit signal is described as a complex function leaving out the constant factor.

Corresponding to the transmitting sequence of the sub-band signals, there exists a time delay between them. According to the TFD shown in Figure 1, the deterministic time delay between the sub-bands corresponds to the frequency offset between them and can be expressed as:

(2) $$\Delta t(k)=(k-1)\frac{\Delta B}{K_{r}}$$

where Δ*B* denotes the frequency interval between the successive bands. Then, the demodulated baseband signal of the point target at the distance *R_t_* in the *k*-th sub-band can be written as

(3) $$\begin{array}{ll} s_{r}(\tau ,k;R_{t})= & \mathrm{rect}\;\left(\frac{\tau -(\frac{2R_{t}}{c}+\Delta t(k))}{T/N}\right)\\ & \exp\;\left\{-j2\pi f_{c}(k)\left(\frac{2R_{t}}{c}+\Delta t(k)\right)+j\pi K_{r}{\left(\tau -\left(\frac{2R_{t}}{c}+\Delta t(k)\right)\right)}^{2}\right\} \end{array}$$

Based on the above result, the signal model of the two modes can be represented in a unified form by Equation (3), enabling a unified synthetic method for both modes.

### 2.2. Error Model

The transmitting and receiving loop of the k-th sub-band signal can be seen as a bandpass filter with the centre frequency *f_c_* (*k*) and bandwidth Δ*B*. Assuming the frequency domain expression of the transmitted signal is *S_t_* (*f_τ_, k*), the received signal in the range frequency domain considering the filter error can be represented by:

(4) $$S_{r}(f_{\tau },k)=S_{t}(f_{\tau },k)H_{f}(f_{\tau },k)$$

where *H_f_* (*f_τ_, k*) denotes the frequency function of the k-th subsystem, *S_r_* (*f_τ_, k*) is the receive signal of the *k*-th sub-band and *f_τ_* denotes the frequency in range dimension. Ideally, *H_f_* (*f_τ_, k*) should be a rectangular window function, covering the desired signal bandwidth exactly In this case, the sub-band signals can be synthesized directly to achieve ultra-high range resolution [11]. However, due to noise in the radar transmitter and receiver circuits, the amplitude-phase characteristics of the filter are not flat as expected, causing amplitude and phase errors of the received signal. Taking the filter error into consideration, the frequency function of the *k*-th filter can then be expressed as:

(5) $$H_{f}(f_{\tau },k)=A_{f}(f_{\tau },k)\exp\{j\Phi_{f}(f_{\tau },k)\}$$

where *A_f_* (*f_τ_, k*) and Φ*_f_* (*f_τ_, k*) are the amplitude and phase characteristics of the *k*-th filter, respectively. In addition to the amplitude-phase error, another error source is the timing error between the sub-bands. As a parallel system composed of N subsystems, its synchronization accuracy is of vital importance. Different radar instruments generate different time delays, inducing timing errors to the multi-sub-band system. Using *ξ* (*k*) to represent the time delay introduced by the instrument in the *k*-th sub-band, the signal with a timing error can be defined by:

(6) $$\begin{array}{ll} s_{\xi }(\tau ,k,R_{t})= & \mathrm{rect}\left(\frac{\tau -(\frac{2R_{t}}{c}+\xi_{t}(k)+\Delta t(k))}{T/N}\right)\exp\left\{-j2\pi f_{c}(k)\left(\frac{2R_{t}}{c}+\xi_{t}(k)+\Delta t(k)\right)\right\}\\ & \exp\left\{j\pi K_{r}{\left(\tau -\left(\frac{2R_{t}}{c}+\xi_{t}(k)+\Delta t(k)\right)\right)}^{2}\right\} \end{array}$$

By transforming Equation (6) into the range frequency domain via the Fourier transform (FT), we obtain:

(7) $$\begin{array}{ll} S_{\xi }(f_{\tau },k,R_{t})= & \mathrm{rect}\left(\frac{f_{\tau }-f_{c}(k)}{B_{\text{sub}}}\right)\\ & \exp\left\{\frac{-j\pi f_{\tau }^{2}}{K_{r}}\right\}\exp\left\{-j2\pi (f_{\tau }+f_{c}(k))\left(\frac{2R_{t}}{c}+\xi_{t}(k)+\Delta t(k)\right)\right\} \end{array}$$

where *B_sub_* is the sub-band bandwidth. The deterministic time delay Δ*t* (*k*) related to the system parameters can be eliminated through a phase multiplication by the following reference function:

(8) $$H_{ct}(f_{\tau },k)=\exp\{j2\pi (f_{\tau }+f_{c}(k))\Delta t(k)\}$$

After compensation of the deterministic time delay between sub-bands, the phase errors left can be written as:

(9) $$\begin{array}{ll} \xi_{p}(f_{\tau },k) & =\exp\{-j2\pi (f_{\tau }+f_{c}(k))\xi_{t}(k)\}\\ & =\exp\{-j2\pi f_{\tau }\xi_{t}(k)\}\exp\{-j2\pi f_{c}(k)\xi_{t}(k)\} \end{array}$$

According to Equations (4), (5), (7) and (9), the signal model in the frequency domain considering the two system errors changes to:

(10) $$\begin{array}{ll} S_{r}(f_{\tau },k) & =S_{t}(f_{\tau },k)H_{f}(f_{\tau },k)\xi_{p}(f_{\tau },k)\\ & =A_{f}(f_{\tau },k)\exp\left\{\frac{-j\pi f_{\tau }^{2}}{K_{r}}\right\}\\ & \;\exp\left\{-j2\pi (f_{\tau }+f_{c}(k))\left(\frac{2R_{t}}{c}+\xi_{t}(k)+\Delta t(k)\right)+j\Phi_{f}(f_{\tau },k)\right\} \end{array}$$

## 3. Effect and Upper Limit of the Timing Error

The effects of amplitude and phase errors have been analysed in detail in [1]. Here, we mainly focus on the effect of the timing error. Considering the timing error only, the constructed signal of an *N*-sub-band system in the frequency domain can be expressed as:

(11) $$S_{rc}(f_{\tau },R_{t})=\sum_{k=1}^{N}S_{t}(f_{\tau },k,T_{t})\xi_{p}(f_{\tau },k)$$

where *S_rc_* (*f_τ_, R_t_*) is the constructed signal. By multiplying *S_rc_* (*f_τ_, R_t_*) with the matched filter and the reference function Equation (8), the constructed signal can be rewritten as follows:

(12) $$S_{rc}(f_{\tau },R_{t})=\sum_{k=1}^{N}\mathrm{rect}\left(\frac{f_{\tau }-f_{c}(k)}{B_{\text{sub}}}\right)\exp\left\{-j2\pi (f_{\tau }+f_{c}(k))\left(\frac{2R_{t}}{c}+\xi_{t}(k)\right)\right\}$$

The inverse Fourier transformation of Equation (12) is given by:

(13) $$\begin{array}{ll} s_{rc}(\tau ,R_{t}) & =\overset{N}{\underset{k=1}{\text{∑}}}Sa\left\{\pi B_{\text{sub}}\left(\tau -\left(\frac{2R_{t}}{c}+\xi_{t}(k)\right)\right)\right\}\\ & \exp\left\{-j2\pi f_{c}(k)\frac{2R_{t}}{c}\right\}\exp\{-j2\pi f_{c}(k)\xi_{t}(k)\} \end{array}$$

Where $Sa(x)=\frac{\sin(x)}{x}$.

For convenience, the linear and constant components of phase errors in Equation (9) are analysed separately. First, the linear component affects the compressed positions of the signals in different sub-bands. If *ξ_t_* (*k*) is larger than the sampling interval, the compressed result for different sub-bands will split into different range cells, which is called the double-vision effect [12]. Since this can be easily identified and avoided by adjusting the range cells, only the timing error less than one sampling interval is considered here. In this case, the same target in different sub-bands is compressed and split into the same range cell, broadening the impulse response width (IRW). Besides, the side-lobes of the compressed pulse will be raised due to the incoherent phase. For the constant phase term, it varies with different sub-bands and results in a phase step at the junction of the neighbouring sub-spectrums. Hence, the constant errors within the sub-band signals can be classified as high order ones for the constructed signal.

**Figure 2:**
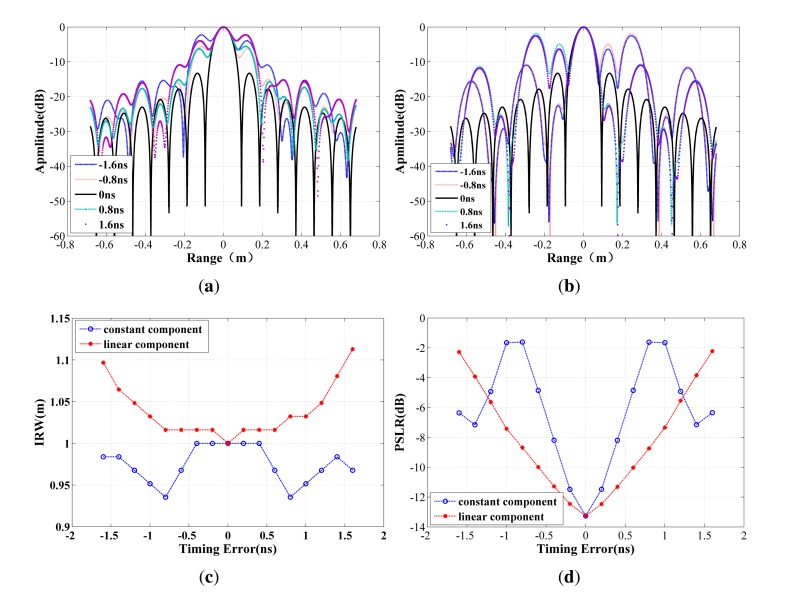
Synthesis results with different time error between sub-bands. (**a**) Impulse response function with linear component of the phase errors; (**b**) impulse response function with constant component of the phase errors; (**c**) impulse response width (IRW) *versus* time error; (**d**) peak side-lobe ratio (PSLR) *versus* time error.

In order to analyse the effect of the two phase error components induced by the timing error, simulations are performed with the parameters listed in Table 1. The impulse response function is shown in Figure 2. The deterioration of the profile is obvious, and the compression result becomes worse with the increase of timing errors. Figure 2a shows that the linear component splits the response and results in symmetric raised side-lobes, which correspond to the deviation of the compressed positions of the signals in different sub-bands. In another case, the asymmetric side-lobes illustrated in Figure 2b demonstrate that the constant component belongs to the class of high order phase errors, which is consistent with our earlier analysis. In Figure 2c,d, the impulse response widths (IRWs) (normalized to the theoretical value) and the peak side-lobe ratio (PSLR) with respect to the timing error are provided. It can be seen that even a nanosecond timing error can degrade the compression quality.

**Figure 3:**
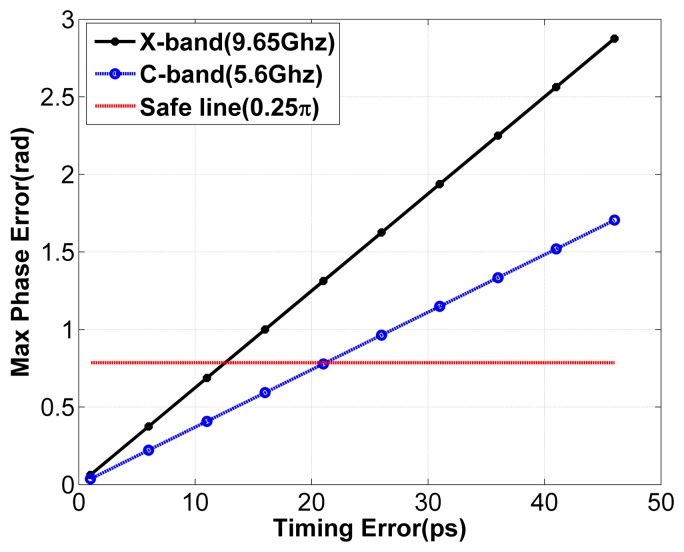
Maximum phase error within the bandwidth *versus* timing error for X-band and C-band SAR systems employing the synthetic bandwidth technique with a slant-range resolution of 0.25 m.

**Figure 4:**
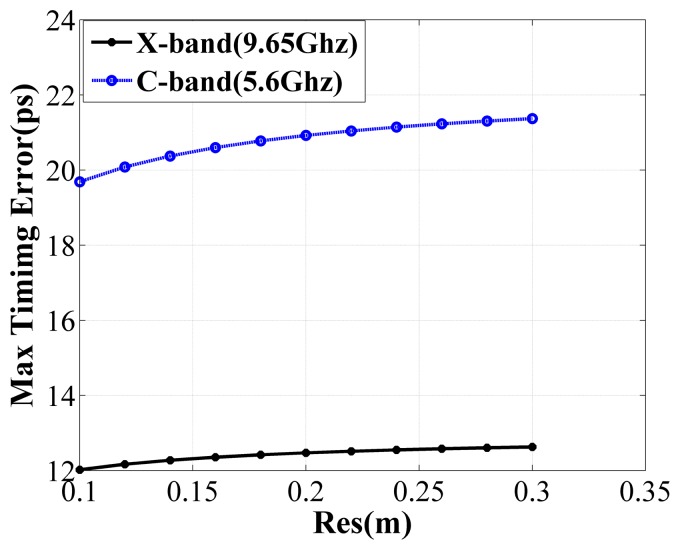
Upper limit of the timing error *versus* the slant-range resolutions for X-band and C-band SAR systems employing the synthetic bandwidth technique.

Next, the requirement on timing accuracy is analysed according to the phase errors introduced by the timing error. By Equation (9), we can see that the timing error between the subsystems will lead to a signal phase error, which increases with frequency. The image quality will be reduced if the phase error exceeds 0.25*π* within the constructed bandwidth [2]. Figure 3 shows the maximum phase errors within the bandwidth caused by different timing errors for X-band and C-band SAR systems employing the synthetic bandwidth technique with a slant-range resolution of 0.25 m, which is the requirement of future applications, such as the TerraSAR Next Generation [13]. As shown by Figure 3, the phase error becomes larger with the increase of timing errors. To limit the phase error under 0.25*π*, the timing error should be less than 12.6*ps* and 21.2*ps* for X-band and C-band SAR systems, respectively. Figure 4 illustrates the upper limit value of timing error for different slant range resolutions. It can be seen that if the synthetic bandwidth technique is applied, the upper limit value of timing error required is at the *ps* level, which is a rather strong requirement on the consistency of the subsystems [14].

## 4. Related Works

Based on the analysis in Section 2, we need to deal with two problems, *i.e.*, timing deviation and the amplitude-phase errors of the system. In the following, we first give a brief review of related works before presenting our approach for constructing the sub-band signals.

### 4.1. Internal Calibration

**Figure 5:**
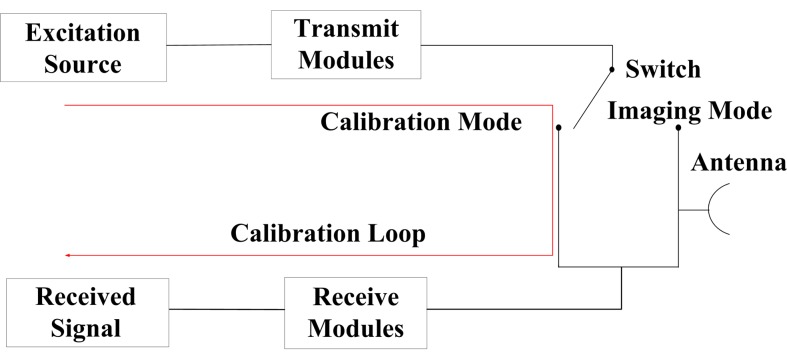
Schematic diagram of an internal calibration system.

Generally, the internal calibration data can be employed to correct the instrument effect on SAR range behaviour [15]. Figure 5 shows the schematic diagram of the internal calibration system [3,8]. In the calibration mode, the excitation source generates a calibration pulse with the same length and bandwidth as the transmitted pulse commanded for the imaging mode. As shown in Figure 5, except for the radar antennas, the calibration signal goes through the transmitting and receiving path directly. Hence, the received calibration pulse is equivalent to the signal of the point target with the slant range being zero. The internal calibration signal is equipped with the feature of the radar system. Therefore, the internal calibration signal can be used to estimate system errors.

### 4.2. The Phase Gradient Autofocus Technique

Phase error is the main component of the errors to be removed in the synthetic bandwidth construction process. As a robust non-parametric approach to phase error estimation, the PGA technique can be used here. It is conventionally applied to reduce phase errors introduced by motion displacements in the azimuth dimension [16] and consists of four processing steps: centre shifting, windowing, phase gradient estimation and iterative correction.

The traditional PGA algorithm begins with the phase-degraded SAR image. By applying the azimuth inverse Fourier transform, the complex image data is transformed into the range compression domain, in which range compression and range cell migration correction (RCMC) has been completed, and the data can be expressed as:

(14) $$F_{j}(l)=|F_{j}(l)|\exp\{2\pi f_{\eta }t_{d}+\phi_{j}(l)+\varphi_{0}\}$$

where *t_d_* is the azimuth time corresponding to the nearest range, *ϕ_j_* (*l*) is the phase error varying with the azimuth bin index *l*, *f_η_* is the azimuth frequency, *j* denotes the range bin and *φ*_0_ represents the initial constant phase.

The first step of PGA is circular shifting by which the linear frequency offset in Equation (14) is removed. Then, the signal is windowed to preserve phase error and to reduce noise. Next, the data are transformed to the range-compressed domain, and Equation (14) becomes:

(15) $${\tilde{F}}_{j}(l)=|F_{j}(l)|\exp\{\phi_{j}(l)+\varphi_{0}\}$$

Then, the phase gradient is calculated and integrated to estimate the phase errors. The maximum-likelihood (ML) kernel of the PGA algorithm is given by:

(16) $$\dot{\varphi }(l)=\arg\left\{\sum_{j}{\tilde{F}}_{j}(l){\tilde{F}}_{j}^{*}(l-1)\right\}$$

where *φ̇*(*l*) is the estimated phase error gradient. Finally, the phase gradient is integrated to obtain the phase errors.

The key to the construction operation of multi-sub-band signals is to correlate the phase of the received signals at each sub-band. PGA has been proven to be a robust method to smooth the phase of the signal in the azimuth direction, and it is possible to apply PGA in the range direction. However, in the process of motion compensation (MOCO), the majority of the motion errors has been eliminated by the navigation measurement, and the PGA method is subsequently applied to estimate the remaining motion errors [17]. If the remaining errors are too large, the PGA technique will not work effectively [12]. Therefore, PGA cannot be implemented on the data in the range dimension directly, and modifications are needed, which forms the basis of our proposed method.

## 5. The Proposed Reconstruction Method

According to the above analysis, we have three challenges for multi-band signal construction. The first one is how to obtain the timing deviation, the precision of which should be up to the *ps* scale; the second one lies in the estimation of amplitude and phase errors of the system. As mentioned in Section 4, internal calibration data can be used to estimate the errors, but there exist residual errors outside the internal loop. The third one is the choice of combination method. The up-sampling operation is needed in the implementation of many construction techniques to avoid aliasing, which increases the data size and the computational load of the system significantly.

**Figure 6:**
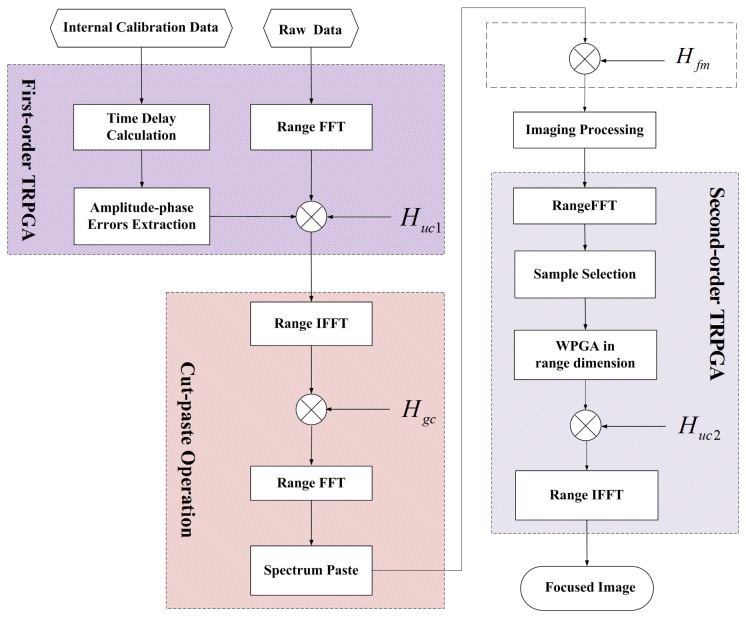
Flow diagram of the proposed method.

We here propose a novel reconstruction method composed of two PGA-based range error correction steps and an improved combination method. Figure 6 shows the flowchart of the proposed synthetic bandwidth construction method. There are three main parts. The first part is the first-order error correction, which estimates errors from the internal calibration data and compensates them for the radar data in the frequency domain. Next is the combination operation by the improved cut-paste method in which the up-sampling operation and the special design of the radar parameters of the traditional combination method [6,10,11] can be avoided. After the above processing, the combined chirp signal is obtained. Then, the imaging algorithm, such as the range Doppler algorithm (RDA), the chirp scaling algorithm (CSA), and the wave-number algorithm (*ωkA*), *etc.*, can be implemented. The last part is the second-order error correction, which removes the residual amplitude and phase errors in the azimuth-compressed domain. In the following, details of the operations are provided.

### 5.1. First-Order Error Estimation and Correction

As the first step of TRPGA for multi-sub-band data construction combination, it accounts for both filter errors and the timing deviation. The fundamental concept of TRPGA is to make a robust estimation of the gradient of the phase errors by exploiting the redundancy of the errors contained in a large number of internal calibration signals. Assuming that the number of internal signals used in this step is *M*, we obtain the internal calibration data matrix as:

(17) $$S_{ic}(k)={\left[s_{ic}(\tau ,k,1),\cdots ,s_{ic}(\tau ,k,m),\cdots ,s_{ic}(\tau ,k,M)\right]}^{T}$$

where *s_ic_* (*τ*, *k*, *m*) is the internal calibration data in the *k*-th sub-band. The dimension of the matrix is *M* × *n_bins_*, where *n_bins_* denotes the range bin number of each sub-band signal. In the matrix, the signal is expressed as a two-dimensional array. The column and row of the matrix can be seen as the range dimension and the azimuth dimension of the conventional PGA, respectively. Because the signal-to-noise ratio (SNR) of the internal signal is much higher than the phase-degraded azimuth signal, iterative correction can be avoided in its implementation. The first-order TRPGA consists of four steps: pulse compression, centre shifting, windowing and phase gradient estimation.

For pulse compression, firstly, performing Fourier transform to *s_ic_* (*τ*, *k*, *m*), we have:

(18) $$\begin{array}{ll} S_{ic}(f_{\tau },k,m) & =A_{f}(f_{\tau },k,m)\exp\left\{\frac{-j\pi f_{\tau }^{2}}{K_{r}}\right\}\\ & =\exp\{-j2\pi (f_{\tau }+f_{c}(k))(\Delta t(k)+{ɛ}_{t}(k,m))+j\Phi_{f}(f_{\tau },k)\} \end{array}$$

where *A*(*f_τ_*,*k*, *m*) denotes the amplitude in the frequency domain and *ε_t_*(*k*, *m*) is the timing error of the *m*-th internal calibration data in the *k*-th sub-band. Then, the quadratic frequency term and the deterministic time delay are removed by multiplying with the function *H_ic_*_1_ (*f_τ_*, *k*) as follows:

(19) $$H_{ic1}(f_{\tau },k)=\exp\left\{\frac{j\tau f_{\tau }^{2}}{K_{r}}+j2\pi (f_{\tau }+f_{c}(k))\Delta t(k)\right\}$$

Then, the signal vector becomes:

(20) $$S_{ic}(f_{\tau },k,m)=A_{f}(f_{\tau },k,m)\exp\{-j2\pi (f_{\tau }+f_{c}(k)){ɛ}_{t}(k,m)+j\Phi_{f}(f_{\tau },k)\}$$

For the centre shifting step, the peak of the compressed signal should be shifted to the centre of the image to remove the linear component in Equation (20). Conventionally, it is implemented by shifting the range cells circularly. Therefore, the precision of the circular shifting operation is at the same level as the time span of a range cell, that is the sampling interval. However, as analysed before, the time accuracy required for multi-sub-band data combination is rather high and much beyond one sampling interval. Therefore, the conventional centre shifting method cannot be applied directly in the first-order error correction.

To improve the accuracy of centre shifting, we can multiply the signal with a linear phase function [2]. First, the instant *ε_t_*(*k*, *m*) is required to be measured precisely, which is the time corresponding to the peak of the compressed pulse, and zero padding is applied to increase the sampling rate [8]. The up-sampling rate *α* is determined by the upper limit of the phase deviation given by Equation (9). According to Equation (9), the phase error increases with frequency for a certain timing deviation. To meet the phase error criterion of 0.25*π* within the total bandwidth, the sampling rate after zero padding needs to meet the following requirement:

(21) $$F_{up}=\alpha f_{s}\geq 8\left(f_{c}+\frac{B_{\text{total}}}{2}\right)$$

where *F_up_* denotes the sampling rate after zero padding and *B_total_* is the bandwidth of the combined signal. After the up-sampling operation, the data are transformed back to time domain, and the time instant *ε_t_* (*k*, *n*) is obtained by measuring the peak position of the compressed pulse. Here, the precision of time is increased by *α* times through the up-sampling operation. Then, accurate centre shifting is implemented by multiplying the centre shifting function to obtain the filter errors. The centre shifting function is given by:

(22) $$H_{ic2}(f_{\tau },k)=\exp\{j2\pi (f_{\tau }+f_{c}(k)){ɛ}_{t}(k,n)\}$$

After this phase multiplication and inverse Fourier transform, the peak of the compressed pulse is moved to the centre, and only the phase error Φ*_f_* (*f_τ_*, *k*) is left in the signal phase.

The next step is windowing. In [16], the window width is determined by the compression result, which is under the assumption that the envelop of the spectrum is approximately rectangular. However, the assumption is invalid, because the magnitude function is an irregular window due to the amplitude error of the filter. To remove the amplitude error, an inverse window function is applied to *S_ic_*(*f_τ_*, *k*, *m*), and it is given by:

(23) $$A_{\text{inv}}(f_{\tau },k,m)=\{\begin{array}{ll} \frac{1}{A_{f}(f_{\tau },k,m)} & \left|f_{\tau }\right|\leq B_{\text{sub}}\\ 0 & \left|f_{\tau }\right|>B_{\text{sub}} \end{array}$$

After this operation, the envelop of the spectrum is approximately rectangular. Then, the signal is transformed to the time domain and windowed. The window width is set to be 50% larger than the length of 10 dB down from the peak of the compressed pulse [16]. After centre shifting and windowing, the phase gradient Φ̇*_f_* (*f_τ_*, *k*) can be estimated by:

(24) $${\dot{\Phi }}_{f}(f_{\tau },k)=\{\begin{array}{ll} 0 & f_{\tau }=0\\ \arg\left\{\sum_{m=1}^{M}S_{ic}(f_{\tau },k,m)S_{ic}^{*}(f_{\tau }-\Delta f_{\tau },k,m)\right\} & \text{other} \end{array}$$

where Δ*f_τ_* is the frequency interval. Let the sampling rate of the sub-band be *f_s_*. Then, $\Delta f_{\tau }=\frac{f_{s}}{n_{\text{bins}}}$. Next, the gradient is integrated to obtain the estimated phase errors Φ*_f_* (*f_τ_*, *k*).

After the error is estimated from the internal calibration data, it comes to error compensation processing, which is applied to the raw data. The first step of the compensation operation is range Fourier transform. Using the first sub-band as the time reference, the uniform amplitude and phase correction function can be written as:

(25) $$H_{uc1}(f_{\tau },k)=H_{uc11}(f_{\tau },k)H_{uc12}(f_{\tau },k)H_{uc13}(f_{\tau },k)H_{uc14}(f_{\tau },k)$$

where:

(26) $$H_{uc11}(f_{\tau },k)=\exp\left\{\frac{j\pi f_{\tau }^{2}}{K_{r}}\right\}$$

(27) $$H_{uc12}(f_{\tau },k)=\exp\{j2\pi (f_{\tau }+f_{c}(k))(\Delta t(k)-\Delta t(1))\}$$

(28) $$H_{uc13}(f_{\tau },k)={\bar{A}}_{\text{inv}}(f_{\tau },k)\exp\{-j\Phi_{f}(f_{\tau },k)\}$$

(29) $$H_{uc14}(f_{\tau },k)=\exp\{j2\pi (f_{\tau }+f_{c}(k))({\bar{ɛ}}_{t}(k)-{\bar{ɛ}}_{t}(1))\}$$

Where ${\bar{A}}_{\text{inv}}(f_{\tau },k)=\frac{\sum_{m=1}^{M}A_{\text{inv}}(f_{\tau },k)}{M}$ and ${\bar{ɛ}}_{t}(k)=\frac{\sum_{m=1}^{M}{ɛ}_{t}(f_{\tau },k)}{M}$. The expectation operation is applied to reduce the effect of white Gaussian noise [8]. In the uniform correction function, the frequency modulation within the sub-band is eliminated by *H_uc_*_11_ (*f_τ_*, *k*). *H_uc_*_12_ (*f_τ_*, *k*) is used to correct the deterministic time delay. The amplitude-phase error and timing error are eliminated by *H_uc_*_13_ (*f_τ_*, *k*) and *H_uc_*_14_ (*f_τ_*, *k*), respectively. After the correction, only the linear phase part corresponding to the time delay for the slant range is left. The task of combination operation is to correlate the linear terms of the different sub-band signals.

### 5.2. The Combination Operation

**Figure 7:**
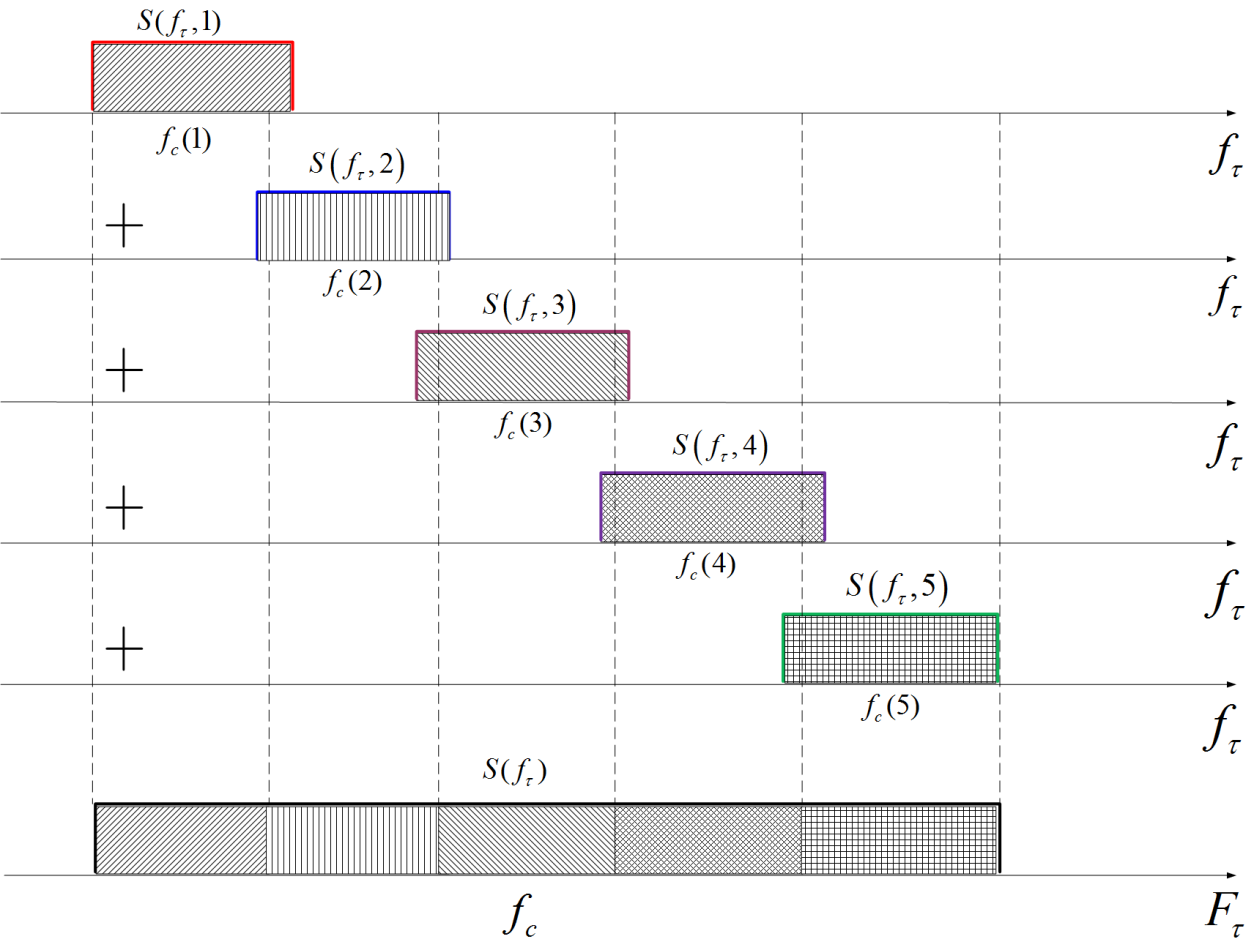
Schematic diagram of the cut-paste method.

As for the combination operation, we propose an improved cut-paste method that combines the sub-band signals in the frequency domain, as shown in Figure 7. In this processing, the sub-band signals are up-sampled uniformly by putting the sub-spectrum data into the appropriate frequency point in the frequency domain, and the up-sampling operation can be avoided for the individual sub-band signals. Assume the frequency vectors of the combined signal and the sub-band signal are respectively *F_τ_* and *f_τ_* (*k*), expressed as:

(30) $$F_{\tau }=\left[f_{c}-\frac{N}{2}f_{s},f_{c}-\frac{N}{2}f_{s}+\frac{f_{s}}{N_{\text{bins}}},\cdots ,f_{c}+\frac{N}{2}f_{s}\right]$$

(31) $$f_{\tau }(k)=\left[f_{c}(k)-\frac{f_{s}}{2},f_{c}(k)-\frac{f_{s}}{2}+\frac{f_{s}}{n_{\text{bins}}},\cdots ,f_{c}(k)+\frac{f_{s}}{2}\right]$$

where *n_bins_*, *N_bins_* is the corresponding number of sampling points of the sub-band signal and the combined signal. The conventional cut-paste method can only be used if each *f_τ_* (*k*) is the subset of *F_τ_*, *i.e.*, the sub-spectrum is a part of the reconstructed spectrum exactly. Otherwise, there is no appropriate position to paste the sub-band signals in the frequency domain. In this case, the system parameters, such as pulse length and A/Dsampling rate, are required to be adjusted [6]. This will increase system complexity, especially considering that many multi-sub-band systems are upgraded from the existing single band system. This problem can be solved through a phase multiplication operation. Let the frequency difference of the *k*-th sub-band be Δ*f_τ_* (*k*). Then:

(32) $$f_{\tau }(k)+\Delta f_{\tau }(k)\subseteq F_{\tau }$$

The frequency gap Δ*f_τ_* (*k*) can be compensated by a phase multiplication in the time domain. The gap correction function is given by:

(33) $$H_{gc}(\tau ,k)=\exp\{-j2\pi \Delta f_{\tau }(k)\tau \}$$

Then, the signal can be transformed back to the frequency domain to implement the cut-paste operation, as shown in Figure 7. After inverse Fourier transform to the merged signal, the high resolution data after range compression is obtained, and many existing imaging algorithms [18–21] can be applied directly. As for the algorithms that do not start with range compression, such as the chirp scaling algorithm [22], frequency modulation needs to be added before being transformed back to the time domain. The frequency modulation phase is given by:

(34) $$H_{fm}(f_{\tau })=\mathrm{rect}\left(\frac{f_{\tau }}{B_{\text{total}}}\right)\exp\left\{-j\pi \frac{f_{\tau }^{2}}{K_{r}}\right\}$$

### 5.3. Second-Order Error Estimation and Correction

The second-order error estimation and correction is applied to remove the residual errors outside the internal loop. The essence of this step is to implement PGA in the range dimension. To have a more effective and efficient estimation, we propose to implement the operation on the azimuth bins with a high signal-to-clutter ratio (SCR). Thereby, a sample selection operation is needed to select the azimuth bins with strong scatters. Firstly, by range Fourier transform, the data are transformed to the azimuth-compressed domain. Then, we select high-quality samples according to the quality PGA (QPGA) selection criteria [23]. After that, the data are transformed back to the time domain, and the qualified bins are selected for error estimation. Assuming the number of qualified azimuth bins is *N_q_*, the selected data can be expressed by an *N_q_* × *N_bins_* matrix ***S****_oc_*.

(35) $$S_{oc}={\left[S_{oc}(\tau ,1),\cdots ,S_{oc}(\tau ,i),\cdots ,S_{oc}(\tau ,N_{q})\right]}^{T}$$

where *S_oc_* (*τ*, *i*) is the data at the *i*-th selected azimuth bin.

In the next step, the strongest scatter is circularly shifted to the centre of the image, after which range Fourier transform is applied to estimate the residual amplitude and phase error in the frequency domain. To reduce the deviation caused by clutters and noise, the weighted least square (WLS) kernel is employed [24,25]. For the WLS estimator, the residual amplitude error *a_wls_* (*f_τ_*) and phase gradient Ψ̇ (*f_τ_*) can be obtained by:

(36) $$a_{\text{wls}}(f_{\tau })=\sum_{i=1}^{N_{q}}\frac{w_{i}}{\sum_{i=1}^{N_{q}}w_{i}}\left|S_{oc}(f_{\tau },i)\right|$$

(37) $$\dot{\Psi }(f_{\tau })=\sum_{i=1}^{N_{q}}\frac{w_{i}}{\sum_{i=1}^{N_{q}}w_{i}}(\arg\{S_{oc}(f_{\tau },i)S_{oc_{k}}^{*}(f_{\tau }-\Delta F_{\tau },i)\})$$

where *S_oc_* (*f_τ_*, *i*) denotes the range frequency domain signal after centre shifting and $\Delta F_{\tau }=\frac{F_{s}}{N_{\text{bins}}}$. The WLS kernel *w_i_* at the *i*-th azimuth bin can be expressed as [24,25]:

(38) $$w_{i}=\frac{1}{\frac{R_{i}}{2}+\frac{5R_{i}^{2}}{24}}$$

where *R_i_* is inverse of the SCR of the azimuth bin, given by:

(39) $$R_{i}=\frac{4}{d_{i}}\left(2c_{i}^{2}-d_{i}-c_{i}\sqrt{4c_{i}^{2}-3d_{i}}\right)$$

with *c_i_*=*E* [|*S_oc_*(*f_τ_*, *i*)|], *d_i_*=*E* [|*S_oc_*(*f_τ_*,*i*)|^2^] and *E*[·] is the expectation operator.

Then, the estimated phase gradient is integrated to acquire phase errors *Ψ_wls_* (*f_τ_*). The second-order amplitude-phase error elimination is performed, with the reference function given by:

(40) $$H_{uc2}(f_{\tau }))=\frac{1}{a_{\text{wls}}(f_{\tau })}\exp\{-j\psi_{\text{wls}}(f_{\tau })\}$$

At last, by applying range inverse Fourier transform to the data, a well-focused image can be obtained. Because only azimuth bins with a high SCR are employed in the estimation, there is no need for iteration, leading to a low-complexity implementation.

## 6. Simulations and Results

To demonstrate the performance and effectiveness of the proposed method for multi-sub-band signal construction, results based on both simulated data and three-sub-band raw data collected through a real system are provided in this section. The data are collected by a radar system operating in strip-map mode. The constructed bandwidth is 880 MHz, and the overlapping bandwidth shared by the neighbouring sub-bands is 10 MHz. The radar system parameters are listed in Table 1.

### 6.1. Results Based on Simulated Data

**Figure 8:**
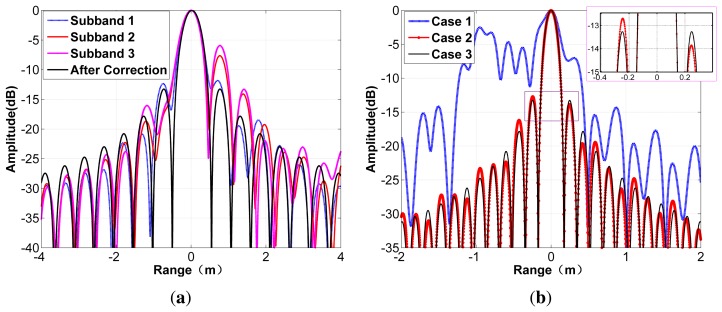
Range compression simulation results: (**a**) compression results of the sub-band signal; (**b**) compression results of synthetic bandwidth for the three cases (1, ignoring timing error; 2, applying the conventional cut-paste method; 3, implementing the improved cut-paste technique).

**Figure 9:**
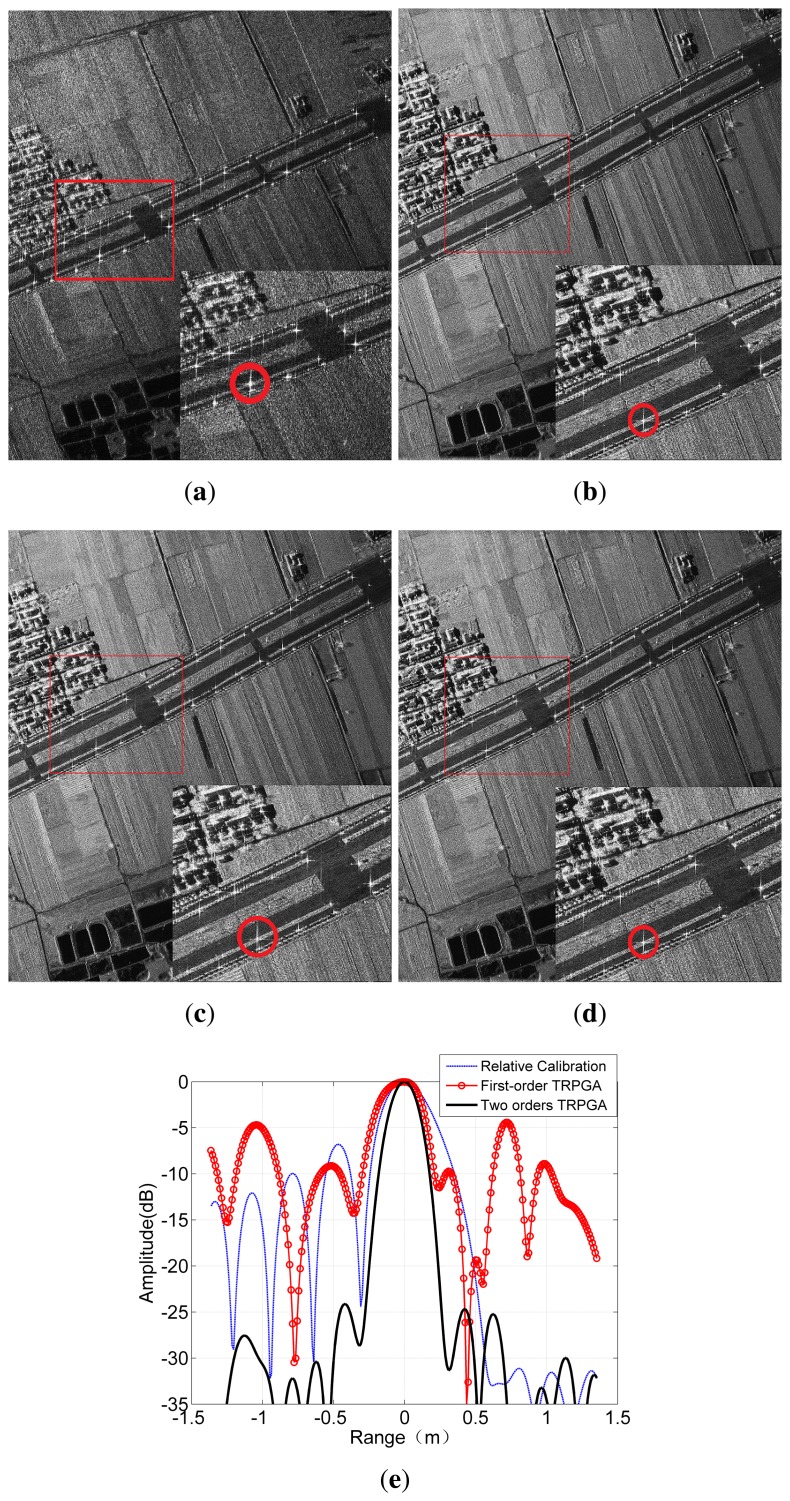
Imaging results based on collected real SAR data: (**a**) imaging result of sub-band signal; (**b**) imaging result of reconstruction with relative calibration; (**c**) imaging result of reconstruction of first-order TRPGA; (**d**) imaging result of reconstruction of two-order TRPGA; (**e**) range profile of point target in (b,c,d).

Simulations are first performed to demonstrate the effectiveness of the first two steps of the proposed method. The filter error is chosen according to the real error calculated from the target point circled in Figure 9a. The timing error between the sub-bands is 4.05 ns and 1.2828 ns, respectively, identical to the timing deviation extracted from the internal calibration data. Figure 8a shows the compressed result when the standard matched filtering is applied to the simulated sub-band data directly. It can be seen that the impulse response function has deteriorated seriously with the side lobes raised and asymmetric, which means that impulse response width (IRW), peak side-lobe ratio (PSLR) and integrated side-lobe ratio (ISLR) cannot meet the imaging requirement any more. After error correction using the first step of the proposed method, the IRW becomes 0.443 m and the PSLR is 13.26 dB, as shown by the solid line. As for the performance of the combination processing, we consider three cases: Case 1, ignoring timing error; Case 2, applying the conventional cut-paste method; and Case 3, implementing the improved cut-paste technique. The blue line represents the construction result without compensation of the timing errors. The timing deviations between the sub-bands split the compressed pulse into several peaks, conforming to the analysis given by Equation (13). After removal of the time deviations, the reconstructed pulse employing the conventional cut-paste method is shown by the red line in Figure 8b. The rise and asymmetry of the side lobes compared with the result processed by the improved cut-paste combination method is obvious, as shown in the enlarged profile. The ideal and measured values of IRW, PSLR and ISLR are listed in the upper part of Table 2. The IRW and PSLR implementing the proposed combination approach are 0.153 m and 13.25 dB, respectively. The consistency between the ideal values and the measured ones indicates that the signals from the three sub-bands have been combined coherently.

### 6.2. Results Based on Collected Raw Data

Now, the raw data collected by a real SAR system with the parameters listed in Table 1 are processed according to the flowchart shown in Figure 6. The image of the one-sub-band echoes is shown in Figure 9a. The vertical direction is the azimuth, and the horizontal direction is the range. Figure 9b shows the combined result of the three sub-band echoes with relative calibration. Compared with Figure 9a, we can see more details of the scene in Figure 9b, such as textures of the house and road, indicating a resolution improvement, that is the bandwidth of the system has been increased effectively. However, the errors within and between the sub-bands have blurred the combined image. To eliminate the errors, the proposed TRPGA is implemented. The imaging results after the first-order TRPGA and the second-order TRPGA are shown in Figure 9c,d, respectively. The image in Figure 9c is clearer after compensation of the errors extracted from the internal calibration data. More detail of the scene, such as the hip roof, can be seen clearly, as a large part of the errors in the system has been eliminated. However, when we zoom into the image, it is clear that the strong points, such as street lamps on the freeway, are defocused in the range dimension due to the existing residual errors. Hence, further processing is required. After implementing the second-order TPRGA, the image quality is improved further, as shown by the zoomed image of Figure 9d.

To demonstrate the improved image quality, the contrast and entropy of the images are measured, and the results are listed in Table 3. The image contrast *C* is defined by [26]:

(41) $$C=\frac{\sqrt{E\{I^{2}(m,n)-E[I^{2}(m,n)]\}}}{E[I^{2}(m,n)]}$$

where *I*^2^ (*m*, *n*) is the image intensity of the pixel (*m*, *n*). The image entropy is used to measure the smoothness of the image and is expressed by [27]:

(42) $$\text{Ent}=\frac{\sum_{m=1}^{M}\sum_{n=1}^{N}\left(\frac{I^{2}(m,n)}{S}\right)}{\ln\left(\frac{I^{2}(m,n)}{S}\right)}$$

where *M* and *N* are the height and width of the measured image and *S* represents the total energy of the image, given by:

(43) $$S=\sum_{m=1}^{M}\sum_{n=1}^{N}I^{2}(m,n)$$

A better combination of the sub-band signals results in larger image contrast and smaller entropy. Therefore, the increase of image contrast and the decrease of entropy from Figure 9b to d correspond to the improvement of image quality. We can also see that the improvement from Figure 9c to d is much more significant than that from Figure 9b to c, which means the first-order error correction can remove most of the system errors, but there still exists residual deviation to be eliminated by the second-order TRPGA.

**Figure 10:**
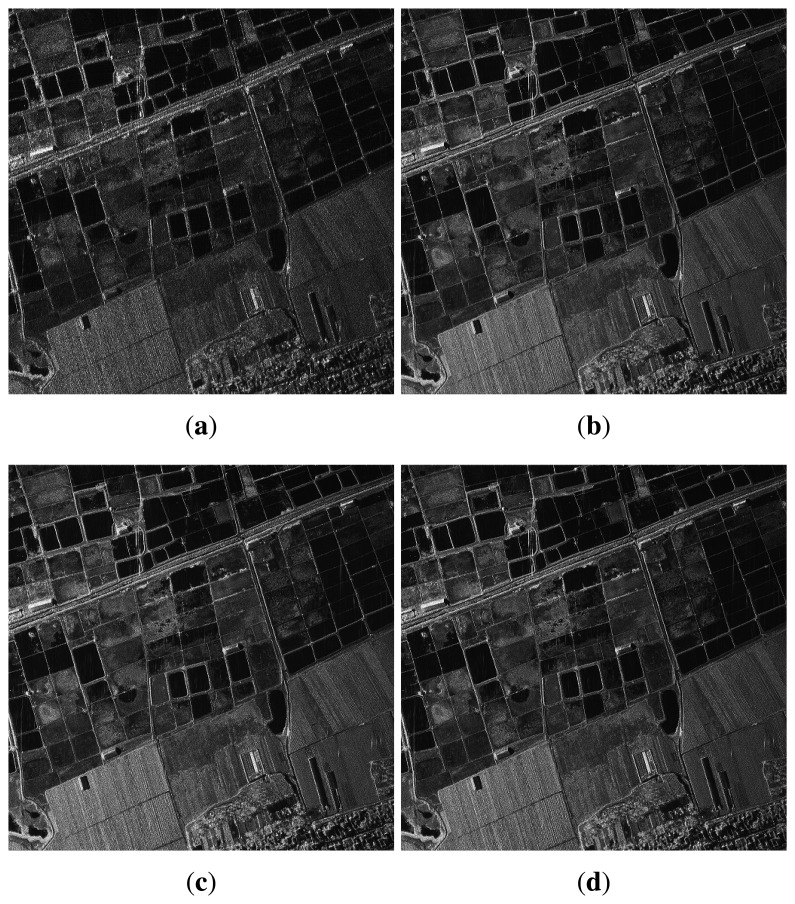
Results of real SAR imaging without strong point target: (**a**) imaging result of sub-band signal; (**b**) imaging result of reconstruction with relative calibration; (**c**) imaging result of reconstruction of first-order TRPGA; (**d**) imaging result of reconstruction of two-order TRPGA.

To show the improvement further, quantitative analysis is performed on the point target circled in Figure 9b–d. The range profiles are shown in Figure 9e in which the Taylor window is applied to suppress the pulse side lobe in image formation. From Figure 9e, it can be seen that the side lobe of the point target is suppressed less than 10 dB after implementing the first-order TRPGA, corresponding to the improvement from Figure 9b to c. However, the profile is far below optimal, and further elimination of the residual error is still necessary. The measured value of IRW, PSLR and ISLR after the implementation of the proposed approach is listed in the lower part of Table 2. The IRW after being processed by the TRPGA is 0.196 m, and the first side lobe is below −25 dB, which is consistent with the theoretical results. Figure 10 is the processing results of the SAR image without strong points. The performance in terms of contrast and entropy is shown in Table 2. The enhancement of image quality shows that the TRPGA method works well, demonstrating the robustness of the proposed technique.

Next, the computational load of the proposed method compared to the one proposed in [28] is analysed. According to [28], the total computational complexity *C_ref_* (only the number of complex multiplications is considered) of the method in [9] is *N_bins_* × *N_q_* × *L* × [(2*N* − 1) log_2_N_bins_ + N (P + 8) − 5], where *L* is the iteration time required and *P* is the highest order of the estimated phase error. As shown in Table 4, the computational load *C_pro_* of the proposed method is *N_bins_* × *N_q_* × (log_2_N_bins_)/2 + 10 × *N_bins_* × *N_q_* [29]. The computation efficiency *ξ_ce_* can be defined as:

(44) $$\xi_{ce}=\frac{C_{\text{ref}}}{C_{\text{pro}}}=\frac{L\times [(2N-1)\log_{2}{\text{N}}_{\text{bins}}+\text{N}(\text{P}+8)-5]}{(\log_{2}{\text{N}}_{\text{bins}})/2+10}$$

As for the image shown in Figure 9, the qualified echo data volume is 32, 768 × 122 (*N_bins_* × *N_q_*); the highest phase error order *P* is six; and the iteration number *L* is five when the method mentioned in [9] is applied. The proposed method spent one minute and 44 seconds to estimate and correct the residual system errors. However, it took 24 minutes and 15 seconds for the algorithm in [9]. According to Equation (44), the computation efficiency is 32, which means that the computational load of our proposed method is significantly lower. It is well worth noticing that from Equation (44), the computation efficiency improves further with the increase of the number of sub-bands, making the proposed method even more efficient for high resolution SAR systems when more sub-bands are required.

## 7. Conclusions

In this paper, a novel bandwidth construction approach has been proposed. It consists of a two-order PGA based range error correction method, named TRPGA, and an improved cut-paste sub-spectrum combination technique. First, the first-order TRPGA estimates the amplitude-phase errors within the sub-bands and the timing error from the internal calibration data. Then, the sub-band signals are combined together by the improved cut-paste technique in which both the up-sampling operation and the special design of radar parameters of the conventional combination method can be avoided. The residual errors are finally eliminated by implementing the second-order TRPGA on the range dimension. The approach accounts for the two main errors of the multi-sub-band system and exploits the redundancy of errors in the set of internal calibration data and the raw data of selected azimuth bins. It is efficient, effective and robust, as demonstrated by imaging results based on both simulated and real data.

## Figures and Tables

**Table 1:** Radar parameters.

**Parameters**	**Symbol**	**Value**	**Units**
Sub-band Number	*N*	3	-
Transmitted Carrier Frequency	*f_c_*	9.63	GHz
Range Resolution	*ρr*	0.15	m
Sub-band Bandwidth	Δ*B*	300	MHz
Sub-band Centre Frequency	*f_c_* (*k*)	9.34/9.63/9.92	GHz
Sub-band Sampling Rate	*f_s_*	320	MHz

**Table 2:** Range impulse response of simulated pulse and real point target.

	**IRW** (m)	**PSLR** (dB)	**ISLR** (dB)
Simulated Pulse Measured Value	0.153	13.25	10.005
Simulated Pulse Theoretical Value	0.151	13.26	10.112
Point Target (weighted) Measured Value	0.196	26.63	20.242
Point Target (weighted) Theoretical Value	0.185	25.55	20.225

**Table 3:** Performance comparison of the images in Figures 9,10.

	**Contrast**		**Entropy**	
	
	Figure 9	Figure 10	Figure 9	Figure 10
Relative Calibration	64.0521	15.0020	14.0498	15.2983
First-order TRPGA	84.3411	18.1717	13.8431	15.1962
Two-order TRPGA	92.6351	20.0582	13.7338	15.1472

**Table 4:** Computational complexity of the proposed TRPGA.

**Operation of the Proposed Method**	**Computational Load**
Range FFT	*N_bins_* × *N_q_* × (*log*_2_*N_bins_*)/2
Sample Selection	2 × *N_bins_* × *N_q_*
WPGA in Range Dimension	7 × *N_bins_* × *N_q_*
Error Compensation	*N_bins_* × *N_q_*

Total Computational Complexity	*N_bins_* × *N_q_* × (log_2_N_bins_)/2 + 10 × *N_bins_* × *N_q_*
